# Compartment-based reconstruction of acquisition-weighted ^31^P cardiac MRSI reduces sensitivity to cardiac motion and scan planning

**DOI:** 10.3389/fphys.2023.1325458

**Published:** 2024-01-19

**Authors:** Andrew Tyler, Moritz J. Hundertmark, Jack J. Miller, Oliver Rider, Damian J. Tyler, Ladislav Valkovič

**Affiliations:** ^1^ Oxford Centre for Clinical MR Research (OCMR), RDM Cardiovascular Medicine, University of Oxford, Oxford, United Kingdom; ^2^ Department of Physiology, University of Oxford, Oxford, United Kingdom; ^3^ School of Biomedical Engineering and Imaging Sciences, King’s College London, London, United Kingdom; ^4^ The MR and PET Research Centres, Aarhus University, Aarhus, Denmark; ^5^ Department of Imaging Methods, Institute of Measurement Science, Slovak Academy of Sciences, Bratislava, Slovakia

**Keywords:** ^31^P, cardiac, spectroscopy, spectroscopy with linear algebra modeling, magnetic resonance spectroscopic imaging, cardiac gating

## Abstract

**Motivation:** ^31^P magnetic resonance spectroscopic imaging (^31^P MRSI) is a powerful technique for investigating the metabolic effects of treatments for heart failure *in vivo*, allowing a better understanding of their mechanism of action in patient cohorts. Unfortunately, cardiac ^31^P MRSI is fundamentally limited by low SNR, which leads to compromises in acquisition, such as no cardiac or respiratory gating or low spatial resolution, in order to achieve reasonable scan times. Spectroscopy with linear algebra modeling (SLAM) reconstruction may be able to address these challenges and therefore improve repeatability by incorporating a segmented localizer into the reconstruction.

**Methods:** Six healthy volunteers were scanned twice in a test–retest procedure to allow quantification of repeatability. Each scan consisted of anatomical localizers and two acquisition-weighted (AW) ^31^P MRSI acquisitions, which were acquired with and without cardiac gating. Five patients with heart failure with a preserved ejection fraction were then scanned with the same ^31^P MRSI sequence without cardiac gating. All ^31^P MRSI datasets were reconstructed with both conventional Fourier transform (FT)-based reconstruction and SLAM reconstruction, which were compared statistically. The effect of shifting the ^31^P MRSI acquisition field of view was also investigated.

**Results:** In the healthy volunteer cohort, the spectral fit of the SLAM reconstructions had significantly improved Cramer–Rao lower bounds (CRLBs) compared to the FT-based reconstruction of non-cardiac gated data, as well as improved coefficients of variability and repeatability. The SLAM reconstruction found a significant difference in the PCr/ATP ratio between the healthy volunteer and patient cohorts, which the FT-based reconstruction did not find. Furthermore, the SLAM reconstruction was less influenced by the placement of the field of view (FOV) of the ^31^P MRSI acquisition in *post hoc* analysis.

**Discussion:** The experimental benefits of the SLAM reconstruction for AW data were demonstrated by the improvements in fit confidence and repeatability seen in the healthy volunteer cohort and *post hoc* FOV analysis. The benefit of SLAM reconstruction of AW data for clinical studies was then illustrated by the patient cohort, which suggested improved sensitivity to clinically significant changes in the PCr/ATP ratio.

## 1 Introduction

^31^P cardiac magnetic resonance spectroscopic imaging (MRSI) is a powerful technique that can be used to measure the ratio of phosphocreatine-to-adenosine triphosphate (PCr/ATP) in the myocardium. The cardiac PCr/ATP ratio provides an insight into the metabolic health of the heart and is strongly predictive of cardiac mortality (Neubauer, 2007). Unfortunately, ^31^P spectroscopy is limited by poor precision and repeatability, limiting its utility in a clinical setting (Bakermans et al., 2017). This is in part due to the low achievable spectral signal-to-noise ratio (SNR), (caused by the low ^31^P concentration in the body and the lower gyromagnetic ratio (*γ*) for ^31^P compared to ^1^H), physiological factors such as cardiac/respiratory motion, and low spatial resolution that lead to spectral contamination artifacts. Long scan times with repeat measurements are typically used to improve SNR, which in turn prevents the use of strategies, such as cardiac gating or respiratory navigators, that would ameliorate physiological motion or the use of higher resolution scans, as both of these would lead to even longer scan times.

A further consideration highlighted by clinicians who currently use MRSI for clinical research is that the strong PCr signal produced by the skeletal muscle in the chest wall indicates that small shifts in FOV (and therefore MRSI voxel position) can significantly alter the measured PCr/ATP ratio. While this is an unwanted source of variability for the measurement, it is also a potential source of bias if a researcher were to, on average, place the voxel slightly closer to or further from the chest wall, particularly in hearts with atypical morphology.

Many technological advances have been made in the field of ^31^P cardiac spectroscopy since its introduction (Bottomley, 1985), including improvements to field strength (Tyler et al., 2008; Rodgers et al., 2014), coil design (Valkovič et al., 2017), and pulse sequence design (Robson et al., 2005). Image reconstruction methodology has also received attention, with compartment-based reconstruction algorithms enabling accelerated acquisitions. Of the methods incorporating compartment-based reconstruction, magnetic resonance spectroscopy with linear algebra modeling (SLAM) (Zhang et al., 2012) and spatial localization with optimal pointspread function (SLOOP) (Von Kienlin and Mejia, 1991) have been applied in several human studies using ^31^P cardiac spectroscopy at 1.5T (Meininger et al., 1999; Beer et al., 2002), 3T (Zhang et al., 2013), and 7T (Tyler et al., 2023). These techniques reduce the number of phase encodes which need to be acquired for adequate spatial resolution; however, they are not currently in widespread use due to the added complexity of adding the required acquisitions (which may need optimizing on a per-participant basis) to clinical research protocols.

All of these techniques incorporate prior information, in the form of an anatomically segmented localizer, into the reconstruction of phase-encoded MRSI data, giving one spectrum per anatomical segment. In the case of SLAM, this is achieved by using linear algebra to reduce the phase-encoded MRSI signal equation to a system of *C* linear equations, where *C* is the number of segmented compartments. Provided that the number of phase encodes *M* is greater than *C*, this system of equations is over-determined, providing the opportunity to reduce *M* and accelerate the scan (Zhang et al., 2012).

Recently, the feasibility of combining SLAM and SLIM (spectral localization by imaging) (Hu et al., 1988) compartment-based reconstruction algorithms with a standard acquisition-weighted (AW) phase-encoded MRSI scan (Tyler et al., 2009) (AW SLAM) to improve SNR and repeatability at 7T compared to Fourier-transform (FT)-based reconstruction of the same data (AW FT-MRS), was demonstrated (Tyler et al., 2023). This AW scan is a commonly used method for 3T clinical research, making it possible to implement AW SLAM while only making minor changes to the clinical research procedure and fully retaining the ability to use the existing Fourier transform-based reconstruction method. Furthermore, we hypothesize that compartment-based reconstruction may ameliorate the effect of cardiac motion due to the larger sensitive region.

In this work, we validate the SLAM reconstruction of AW ^31^P cardiac acquisitions at 3T for clinical research, comparing it to our existing FT-MRS reconstruction technique across a healthy volunteer repeatability study and clinical research data. The repeatability study also consisted of ^31^P scans both with and without cardiac gating to allow the motion robustness of the SLAM technique to be assessed. The repeatability study dataset was additionally reconstructed with different shifted FOVs to assess the impact of errors when setting up the acquisition. For all experiments, PCr/ATP ratio, PCr SNR, PCr/ATP ratio Cramer–Rao lower bounds, and coefficients of repeatability (CoR) and variation (CoV) were calculated to allow quantitative comparison.

## 2 Materials and methods

**FIGURE 1:**
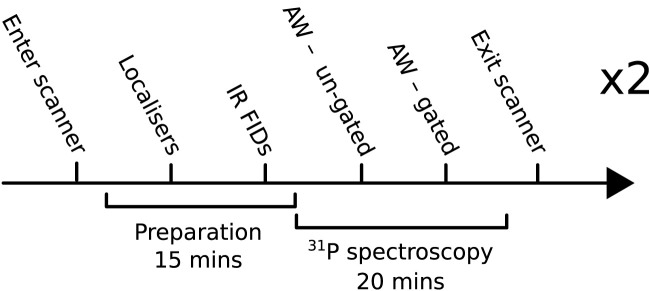
Overview of the protocol used for the main repeatability study. IR FID, inversion-recovery-free induction decay; AW, acquisition-weighted.

The experiments reported in this study consist of a healthy volunteer repeatability study and an applied study. For the repeatability study, six healthy volunteers (2F/4M, age = 31 ± 8 years, no history of cardiovascular disorders, metabolic syndrome, or any other chronic disease) were scanned twice with an AW phase-encoding scheme. Each scan consisted of ^1^H localizers and two ^31^P acquisitions with and without cardiac gating (denoted G and UG, respectively). An overview of the protocol is shown in Figure 1. The applied study included five patients (2F/3M, BMI = 36 ± 5 kg/m^2^, age = 69 ± 9 years) with heart failure with a preserved ejection fraction (HFpEF) who were each scanned once without cardiac gating as part of a clinical study where adding an additional gated acquisition was not feasible.

All volunteers provided informed consent for this study, which was approved by the Research Ethics Committee and was consistent with the International Council for Harmonisation of Technical Requirements for Pharmaceuticals for Human Use (ICH) Guidelines for Good Clinical Practice (GCP).

### 2.1 Data acquisition

All acquisitions were performed using a Siemens 3T TIM Trio whole-body MRI scanner (Siemens Healthineers, Erlangen, Germany) with volunteers positioned in the prone position. The coil consisted of a ^1^H/^31^P dual-tuned 26 × 28 cm transmit/receive loop and a 2 × 12 × 15 cm butterfly ^31^P receive pair (Tyler et al., 2009). Two-chamber, four-chamber, short axis (FOV = 400 × 340 mm), and MRS-matched ^1^H images were acquired with a fast low-angle shot (FLASH) sequence (Hasse et al., 1986). The coil position was determined from images showing the location of four cod-liver oil capsules and one central phenylphosphonic acid fiducial embedded in the coil housing during imaging, and the transmit flip-angle was calculated from a series of inversion-recovery-free induction decay acquisitions of the central fiducial as previously described (Tyler et al., 2009). The standard “tune-up” shim setting was used for all ^31^P acquisitions.

**FIGURE 2:**
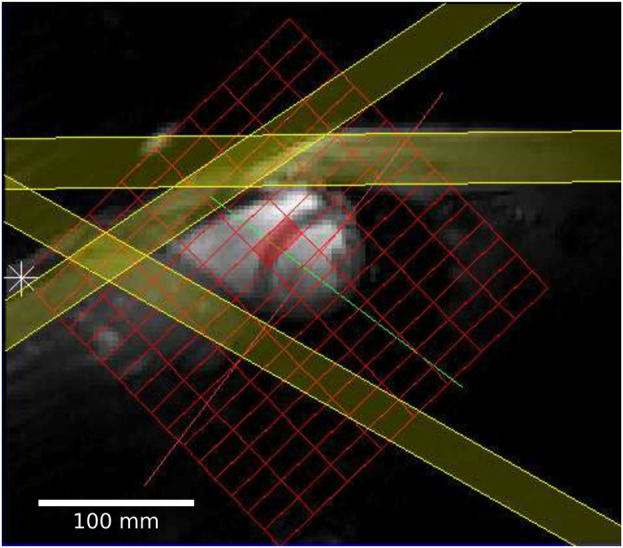
Short axis localizer showing the FT-MRS grid in red and three PCr saturation bands in yellow; two of the bands saturate the PCr signal from the chest, and the third saturates the liver close to the heart.

All ^31^P acquisitions consisted of a UTE-CSI sequence (Robson et al., 2005) with a shaped RF-pulse, numerically optimized for excitation homogeneity and NOE enhancement. The excitation was centered at −250 Hz relative to the PCr resonance between the *γ*-ATP and *α*-ATP resonances to provide uniform excitation of the ^31^P spectrum across a spectral width of approximately 1.5 kHz (Tyler et al., 2009). Cardiac gating (where used) was prospective and triggered the acquisition to coincide with diastasis. Three saturation slabs (thickness = 25 mm) were used to null the signal from the chest wall and liver (Figure 2). All ^31^P acquisitions were acquisition-weighted (10 averages at the center of k-space) and had a FOV of 240 × 240 × 200 mm and a nominal resolution of 30 × 15 × 25 mm. In the absence of cardiac gating, the acquisition had a duration of 10.5 min with a nominal TR of 0.9 s. The total time for one repeat of the protocol was approximately 35–40 min, which was repeated twice per participant.

### 2.2 Data analysis

**FIGURE 3:**
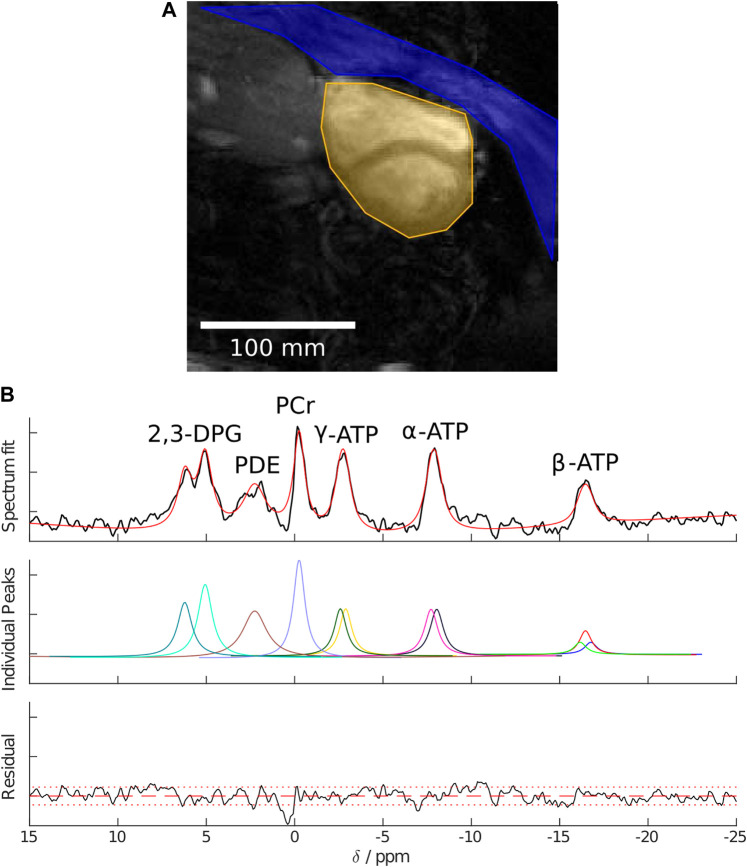
**(A)** Example segmentation masks showing the chest wall in blue, heart in yellow, and “other” in greyscale. **(B)** Example cardiac spectra for a healthy volunteer reconstructed using the SLAM algorithm, AMARES fitted peaks, and fit residual.

In each case, the same raw ^31^P dataset was reconstructed with both SLAM and FT-MRS algorithms, according to Tyler et al. (2023). For the SLAM reconstruction, the MRS-matched anatomical localizer was segmented into three compartments (heart, chest wall, and others), and the SLAM reconstruction algorithm was used to produce one spectrum per compartment. For the FT-MRS reconstruction, the raw dataset was Fourier-transformed in each spatial dimension before a time-domain Fourier transform was applied to give one spectra per voxel. The mid-septal voxel (Figure 2) was then chosen and used for further analysis to minimize spectral contamination (Ellis et al., 2019). An example segmentation mask showing the different compartments used for the SLAM reconstruction is shown in Figure 3, with the corresponding fitted heart compartment spectra underneath.

The spectra produced by both the SLAM and FT-MRS reconstruction algorithms were post-processed identically. In each case, the spectra were phase-corrected (zeroth and first order), apodized, and fitted with the Oxford spectroscopy analysis (OXSA) implementation of AMARES (Purvis et al., 2017). The PCr/*γ*-ATP ratios calculated from the fit were blood- and saturation-corrected (Rodgers et al., 2014).

The mean, Cramer–Rao lower bounds (CRLBs), coefficient of repeatability (CoR), and coefficient of variability (CoV) were calculated for the PCr/ATP ratio. SNR of the PCr resonance was also calculated as the amplitude of the PCr resonance divided by the standard deviation of the last 104 points in the reconstructed spectrum (outside of the region excited by the sequence). CoR was defined as (Ellis et al., 2019)

$$CoR={SD}_{\text{intrasubject}}\times 1.96,$$ (1)

where SD_intrasubject_ is the standard deviation (SD) of the signed difference in the PCr/ATP ratio between scans of the same subject with the same technique. CoV was defined as SD of a measure across all scans (both sessions) of the same technique divided by the mean value of the measure. All comparisons were made to the UG AW FT-MRS method with pairwise two-tailed Wilcoxon signed rank tests at a 5% significance level.

### 2.3 Simulation of the FOV shift

As an addition to the repeatability study, FOV of the ^31^P datasets was shifted toward and away from the chest wall by ± 1 voxel in increments of 0.5 voxels. This simulates both the effect of incorrect placement of the FT-MRS mid-septal voxel during acquisition and subject motion between acquisition of the ^1^H localizer and ^31^P. The FOV-shifted data were reconstructed using the SLAM and FT-MRS methods. The SLAM segmentation masks were subsequently corrected for this shift to the ^31^P FOV and reconstructed.

## 3 Results

### 3.1 Healthy volunteer repeatability study

#### 3.1.1 UG AW FT-MRS

**FIGURE 4:**
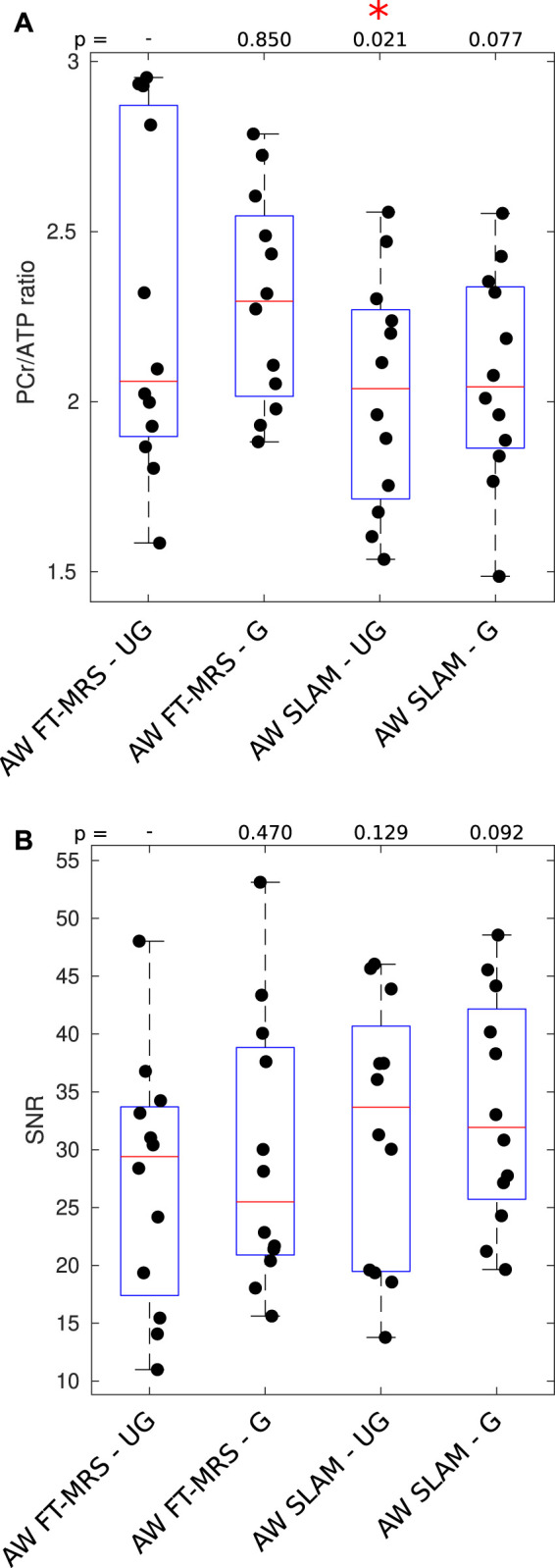
Box plot showing PCr/ATP values **(A)** and PCR SNR **(B)** for each acquisition; median and IQR are indicated by box. * indicates significant difference for the UG AW FT-MRS method (Wilcoxon signed-rank paired, *α* = 0.05). PCr/ATP values are in the expected range (Table 3).

The PCr/ATP ratio and PCr SNR of each spectrum are shown as a box plot in Figure 4. The mean PCr/ATP ratio and PCr SNR of the healthy volunteer cohort as well as the PCr linewidth, PCr/ATP CRLB, CoV, and CoR are given in Table 1. The PCr/ATP ratio was 2.27 ± 0.50, PCr SNR was 27.2 ± 10.8, PCr FWHM was 21.9 ± 5.8 Hz, and CRLB was 11.6% ± 6.8%.

**TABLE 1:** Mean values of the experimental results derived for each method, and errors are given as ± 1 SD.

Algorithm	Gating	PCr/ATP	PCr SNR	PCr FWHM (Hz)	CRLB (%)	CoR	CoV
FT-MRS	UG	2.27 ± 0.50	27.2 ± 10.8	21.9 ± 5.8	11.6 ± 6.8	1.02	0.22
	G	2.30 ± 0.31	29.4 ± 11.7	20.5 ± 5.8	11.7 ± 8.6	0.77	0.14
SLAM	UG	2.03 ± 0.34*	31.6 ± 11.4	27.8 ± 6.9*	7.81 ± 3.29*	0.66	0.17
	G	2.07 ± 0.31	33.4 ± 9.8	25.9 ± 7.4*	7.92 ± 3.56*	0.67	0.15

* indicates significant difference for the UG AW FT-MRS method (Wilcoxon signed-rank paired, α = =0.05). UG indicates that the acquisition was not cardiac-gated and G indicates that the acquisition was cardiac-gated, with the readout timed to coincide with diastasis.

#### 3.1.2 Gated AW FT-MRS

The results of the gated AW FT-MRS method are shown in Figure 4. The mean PCr/ATP ratio, CRLB, CoV, and CoR, as well as PCr SNR, are shown as a part of Table 1. The gated AW FT-MRS method did not have significantly different PCr/ATP ratios (2.30 ± 0.31, *p* = 0.850), PCr SNR (29.4 ± 11.7, *p* = 0.470), PCr FWHM (20.5 ± 5.8 Hz, *p* = 0.052), or CRLB (11.7 ± 8.6, *p* = 0.569) to the UG AW FT-MRS method; however, both the CoV and CoR were lower, indicating improved repeatability.

#### 3.1.3 SLAM reconstruction

Box plots displaying the PCr/ATP ratio and PCr SNR for the SLAM reconstruction of the gated and ungated acquisitions are shown in Figure 4. The mean statistics for each method are shown in Table 1. Both UG and G AW SLAM had significantly lower CRLBs compared to the UG AW FT-MRS (UG SLAM 7.81% ± 3.29%, *p* = 0.0005, G SLAM 7.92% ± 3.56%, *p* = 0.0010). UG AW SLAM had a significantly lower PCr/ATP ratio than the UG AW FT-MRS (2.03 ± 0.34 vs. 2.27 ± 0.50, *p* = 0.021), although the medians were similar, UG AW SLAM = 2.04 vs. UG FT-MRS = 2.06. Both UG and G AW SLAM had significantly higher PCr linewidths compared to UG AW FT-MRS (UG SLAM 27.8 ± 6.9 Hz, *p* = 0.0049, G SLAM 25.9 ± 7.4 Hz, *p* = 0.0210).

### 3.2 FOV shift

**FIGURE 5:**
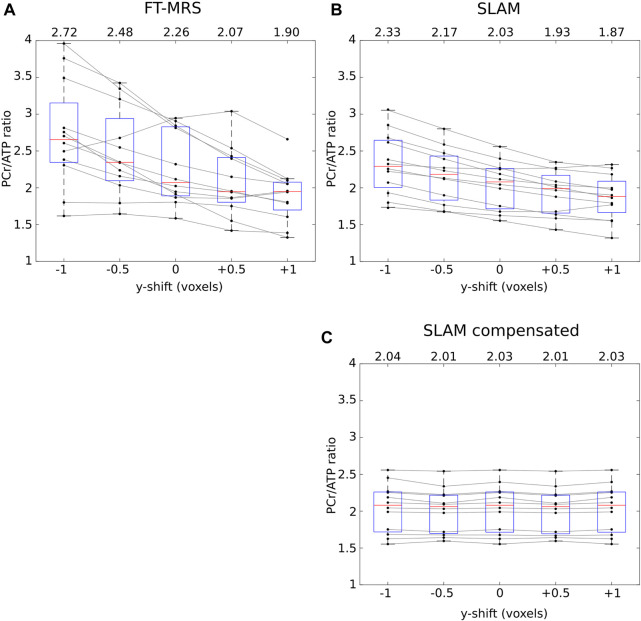
Each panel shows how the reconstructed PCr/ATP ratios of the repeatability dataset for each method varies as the data are shifted in the *y*-direction (approximately anterior–posterior, up–down on Figure 3). The mean PCr/ATP of each shifted dataset is shown above the corresponding box, and the points corresponding to a single scan are joined by gray lines. The SLAM reconstruction **(B)** shows markedly less change in the PCr/ATP ratio than the FT-MRS reconstruction **(A)**. For the SLAM compensated method **(C),** the location of the compartmentalization mask is compensated by the same amount as the shift applied to the data, demonstrating that the SLAM method is insensitive to the exact position the FOV is placed.

The effect of shifting the FT-MRS mid-septal voxel/FOV of ^31^P acquisition FOV on the reconstructed PCr/ATP values with each method is shown in Figure 5. The change in the mean PCr/ATP ratio across the two-voxel shift range is 0.82 for FT-MRS and 0.46 for SLAM. When the SLAM segmentation mask is compensated for the movement in FOV (i.e., the segmentation mask is corrected for ^31^P with shift), the change in the PCr/ATP values was minimal.

### 3.3 Patient study

**FIGURE 6:**
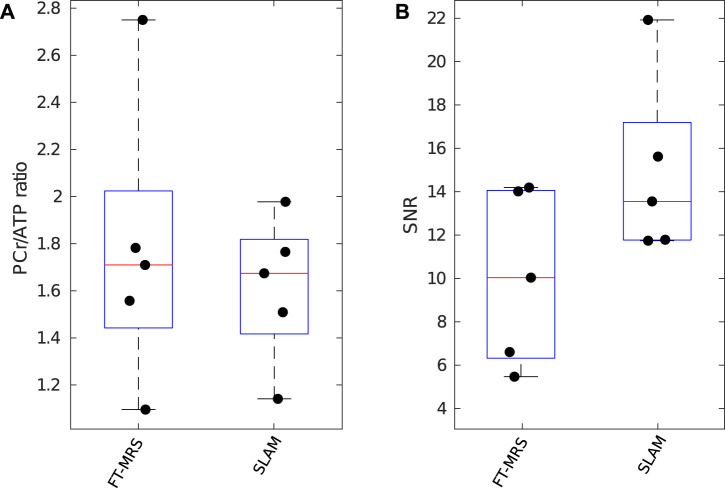
Box plot showing PCr/ATP values **(A)** and PCR SNR **(B)** for each reconstruction method applied to the clinical research HFpEF dataset; median and IQR are indicated by box. PCr/ATP values are lower than those of the healthy control volunteers.

Box plots displaying the computed PCr/ATP ratio and PCr SNR for SLAM and FT-MRS reconstructions of the clinical HFpEF dataset are shown in Figure 6. The results for each method are shown in Table 2. Both methods had similar PCr/ATP ratios, with a lower range of values than the healthy controls. There was no significant difference in PCr SNR between the FT-MRS and SLAM reconstruction (pairwise two-tailed Wilcoxon, *p* = 0.3125), although the SLAM SNR was 48% higher in this small cohort. Additionally, when comparing the healthy volunteer and HFpEF datasets, the SLAM reconstruction showed a significant reduction in PCr/ATP in the HFpEF participants (Welch’s *t*-test, *p* = 0.0233), whereas the FT reconstruction did not (*p* = 0.1606).

**TABLE 2:** Mean values of the experimental results derived for each reconstruction method applied to the clinical research HFpEF (heart failure with a preserved ejection fraction) dataset. Errors are given as ± 1 standard deviation.

Method	PCr/ATP	PCr SNR	CRLB (%)	CoV
AW FT-MRS	1.78 ± 0.61	10.1 ± 4.1	27.5 ± 13.7	0.34
AW SLAM	1.61 ± 0.27	14.9 ± 4.2	17.3 ± 6.6	0.19

## 4 Discussion

In this work, we investigated the utility of AW-SLAM for clinical research at 3T. Our healthy volunteer repeatability study demonstrated the utility of SLAM reconstruction to improve fit confidence and repeatability over a conventional FT-MRS reconstruction, as well as its increased robustness to discrepancies in the FOV position between the localizer and ^31^P acquisition and its potential for removing experimental bias. The patient volunteer cohort then demonstrated the experimental benefits of AW-SLAM for clinical trials by finding a significant difference in the PCr/ATP ratio between five HFpEF patients and the healthy volunteer repeatability cohort, which AW FT-MRS did not find.

### 4.1 Healthy volunteer repeatability study

#### 4.1.1 UG AW FT-MRS

The measured PCr/ATP ratio (2.27 ± 0.50) is at the upper end of literature values (Table 3); however, given the lack of morbidities and good health of the volunteer cohort, it is credible. When compared to a previous repeatability study performed in our center (Tyler et al., 2009), using the same hardware but with cardiac gating and a higher resolution (31 min) acquisition, the PCr/ATP ratio is comparable, PCr/ATP = 2.27 ± 0.50 (this work) and 2.07 ± 0.38 or 2.14 ± 0.46 (Tyler et al., 2009). The coefficients of repeatability and variation were closely comparable, CoV = 0.22 (this work) and 0.18 (Tyler et al., 2009) and CoR = 1.0 (this work) and 1.1 (Tyler et al., 2009), suggesting that the data presented in this study are broadly representative of the capabilities of the technique.

**TABLE 3:** Selected literature PCr/ATP values.

Study	Field strength	Technique	PCr/ATP ratio
Ellis et al. (2019)	7	FT-MRS	1.71 ±0.65
Rodgers et al. (2014)	7	FT-MRS	2.1 ±0.3
Tyler et al. (2009)	3	FT-MRS	2.07 ±0.38
			2.14 ±0.46
Lamb et al. (1996)	1.5	3D ISIS	1.31 ±0.19

#### 4.1.2 Effect of gating

The PCr/ATP ratio, PCr SNR, and PCr/ATP CRLB of G AW FT-MRS were very close in value to UG AW FT-MRS (<10%) with no significant differences; however, there were larger differences in CoV and CoR, with the G AW FT-MRS method performing better. This suggests that acquiring data over all cardiac phases does not introduce systematic bias compared to a gated acquisition for AW FT-MRS but introduces variance into the result. One potential explanation for this could be that for an UG acquisition, over many phase encodes across multiple scans, the mid-septum of the heart will be, on average, in the position determined by the localizer; however, for the critical central phase encodes in an individual scan, this is not necessarily true, adding variance compared to the gated scan (Wampl et al., 2021). Although not the main focus of the work presented in this study, this result suggests that cardiac gating of ^31^P FT-MRSI in the heart improves repeatability and is worthy of further study.

For the SLAM reconstruction, the same improvements in CoR and CoV relative to the ungated acquisition were not seen, despite the gated acquisition counteracting the effect of cardiac motion. This could be due to the increase in the size of the sensitive region of SLAM relative to AW FT-MRS, which could mean that the septum of the heart, to a greater extent, would have stayed within the larger area during the heartbeat.

#### 4.1.3 SLAM reconstruction

The AW SLAM reconstructions had PCr/ATP ratios within the range of literature values (Table 3). UG AW SLAM had a significantly lower PCr/ATP ratio (2.03 ± 0.34) than UG AW FT-MRS (2.27 ± 0.50); however, the UG AW FT-MRS is at the top end of literature values, while the ratio recorded by AW SLAM was comfortably within the range of literature values. Furthermore, this reduction in PCr/ATP suggests that the risk, where lower CRLBs can imply spectral contamination from the strong, high-SNR PCr signal of the chest wall (which shifts the measured PCr/ATP ratio higher), is unlikely to be present.

The reduction in both CoR and CoV of the SLAM reconstructions compared to the UG AW FT-MRS may, in part, have been due to the significantly lower PCr/ATP CRLBs, indicating improved confidence in the fit. However, the reduction in CoR and CoV of G AW FT-MRS compared to UG AW FT-MRS, coupled with the similarity in CoV between G AW FT-MRS and G AW SLAM, suggests that cardiac motion could be a more important factor. Reassuringly, this also implies that the reduction in CoV seen with SLAM was a real reduction in the experimental error caused by cardiac motion and not merely a reduction in sensitivity to changes in the PCr/ATP ratio.

Both UG and G SLAM had significantly higher linewidths (PCr FWHM) than UG FT-MRS; this may be due to the larger sensitive region of the acquisition, which would encompass a larger range of B_0_ values within the scanner. This could be problematic for detecting some species, such as inorganic phosphate, in crowded regions of the ^31^P spectrum; however, the significantly lower CRLBs for UG FT-MRS suggest that the linewidth is not overly deleterious to the method. Furthermore, the use of subject-specific shim strategies (Ellis et al., 2019) could reduce the PCr linewidth by improving B_0_ homogeneity over the whole heart, at the expense of additional exam time.

The remaining spread in the PCr/ATP ratios of G and UG SLAM is likely due in part to biological variation in the true PCr/ATP ratio of the volunteers, breathing motion, and bulk volunteer motion. Although biological variation is intrinsic to cardiac ^31^P MRSI, the effect of breathing and bulk motion could be addressed through refinements to the technique, including shorter scan times and diaphragmatic navigator-based gating strategies.

Overall, our data point to two important findings. First, the SLAM reconstruction method provides improved performance over FT-MRS reconstruction with or without cardiac gating. Second, while cardiac gating improves repeatability for both SLAM and FT-MRS, the SLAM reconstruction provides most of the improvements to the repeatability of cardiac gating without the time penalty this would entail.

### 4.2 FOV shift

The simulated effect of shifting the ^31^P acquisition FOV demonstrated that the FT-MRS reconstruction was highly sensitive to the position of the mid-septal voxel, with a shift of half a voxel (7.5 mm) toward the chest wall, resulting in an increase in the PCr/ATP ratio of 0.22 across the patient cohort. In comparison, the SLAM reconstruction showed lower sensitivity, with the same shift causing an increase in the PCr/ATP ratio of 0.14, potentially explaining the reduction in CoR of the SLAM reconstruction compared to FT-MRS. This is particularly advantageous for SLAM as the impact of patient motion between the localizer and ^31^P acquisition is reduced, a significant concern for patient cohorts, particularly when scanned in the less-comfortable prone position.

In addition, the consistency of the mean PCr/ATP ratio when the FOV shift is compensated (i.e., the segmentation mask is correctly defined for the FOV acquired) for the SLAM reconstruction implies that so long as the segmentation mask is correctly defined during analysis, the SLAM method is relatively immune to errors when defining the ^31^P FOV on the scanner. Excitingly, this could allow operator bias to be eliminated almost entirely in the future, for instance, using a machine learning algorithm (Chen et al., 2020) to perform the segmentation after acquisition, knowing that the positioning of FOV has little impact on the final result.

### 4.3 Patient scans

Both the SLAM and FT-MRS reconstructions of the HFpEF patient cohort had plausible mean PCr/ATP ratios (1.61 ± 0.27 and 1.78 ± 0.61, respectively), when compared to Burrage et al. (2021) who recorded 1.66 ±0.22 for 14 HFpEF patients. In the FT-MRS reconstruction, significant differences in PCr SNR and PCr/ATP CRLB were not found; however, together with the CoV, they showed non-significant improvement, which is supported by the healthy volunteer cohort results.

The benefit of using the compartmentalized methods for these participants can be seen by calculating the sample size required for an experiment, investigating if HFpEF affects the PCr/ATP ratio compared to healthy controls and having a statistical power of 0.8 (*t*-test, *α* = 0.05). Using the means and standard deviations tabulated in Tables 1, 2 UG AW FT-MRS would require 34 participants in total, whereas SLAM would require 16 participants. This is reflected in the significant difference in the PCr/ATP ratio between the healthy volunteer repeatability study cohort and HFpEF cohort, seen for the SLAM reconstruction but not the FT-MRS reconstruction.

## 5 Conclusion

In this work, we showed that the SLAM reconstruction of the AW FT-MRS data improved both fitting confidence and repeatability compared to the FT-MRS reconstruction. We also showed that cardiac gating during acquisition improved repeatability for both FT-MRS and SLAM reconstructions, although the UG SLAM method delivered most of the benefits of gating, allowing for shorter scan times. Additionally, we demonstrated that the SLAM reconstruction was independent of the exact FOV acquired, allowing automated segmentation during reconstruction compared to setting the mid-septal voxel at scan time.

The advantages of using SLAM reconstruction were further illustrated by the five volunteer HFpEF datasets, which clearly demonstrate the clinical advantage of the reconstruction technique in a highly challenging patient population by achieving statistical significance against the healthy volunteer dataset, unlike the FT-MRS reconstruction method when applied to the same data.

Overall, the findings of this work make a compelling case for compartment-based reconstruction of clinical ^31^P cardiac spectroscopic research scans.

## Data Availability

The raw data supporting the conclusion of this article will be made available by the authors without undue reservation.

## References

[B1] Bakermans A. J. Bazil J. N. Nederveen A. J. Strijkers G. J. Boekholdt S. M. Beard D. A. (2017). Human cardiac 31P-MR spectroscopy at 3 Tesla cannot detect failing myocardial energy homeostasis during exercise. Front. Physiology 8, 939. 10.3389/fphys.2017.00939 PMC5712006 29230178

[B2] Beer M. Seyfarth T. Sandstede J. Landschütz W. Lipke C. Köstler H. (2002). Absolute concentrations of high-energy phosphate metabolites in normal, hypertrophied, and failing human myocardium measured noninvasively with 31P-SLOOP magnetic resonance spectroscopy. J. Am. Coll. Cardiol. 40 (7), 1267–1274. 10.1016/S0735-1097(02)02160-5 12383574

[B3] Bottomley P. A. (1985). Noninvasive study of high-energy phosphate metabolism in human heart by depth-resolved ^31^p nmr spectroscopy. Science 229, 769–772. 10.1126/science.4023711 4023711

[B4] Burrage M. K. Hundertmark M. Valkovič L. Watson W. D. Rayner J. Sabharwal N. (2021). Energetic basis for exercise-induced pulmonary congestion in heart failure with preserved ejection fraction. Circulation 144, 1664–1678. 10.1161/CIRCULATIONAHA.121.054858 34743560 PMC8601674

[B5] Chen C. Qin C. Qiu H. Tarroni G. Duan J. Bai W. (2020). Deep learning for cardiac image segmentation: a review. Front. Cardiovasc. Med. 7, 25. 10.3389/fcvm.2020.00025 32195270 PMC7066212

[B6] Ellis J. Valkovič L. Purvis L. A. B. Clarke W. T. Rodgers C. T. (2019). Reproducibility of human cardiac phosphorus MRS (31P-MRS) at 7T. NMR Biomed. 32, e4095. 10.1002/nbm.4095 30924566 PMC6546607

[B7] Hasse A. Frahm J. Matthaei D. Hanicke W. Merboldt K. D. (1986). FLASH imaging. rapid NMR imaging using low flip-angle pulses. J. Magnetic Reson. 67, 258–266. 10.1016/0022-2364(86)90433-6 22152368

[B8] Hu X. Levin D. N. Lauterbur P. C. Spraggins T. (1988). SLIM: spectral localization by imaging. Magnetic Reson. Med. 8, 314–322. 10.1002/mrm.1910080308 3205158

[B10] Lamb H. J. Doornbos J. den Hollander J. A. Luyten P. R. Beyerbacht H. P. van der Wall E. E. (1996). Reproducibility of human cardiac 31P-NMR spectroscopy. NMR Biomed. 9, 217–227. 10.1002/(SICI)1099-1492(199608)9:5<217::AID-NBM419>3.0.CO;2-G 9068003

[B11] Meininger M. Landschu W. Beer M. Seyfarth T. Horn M. Pabst T. (1999). Concentrations of human cardiac phosphorus metabolites determined by SLOOP 31P NMR spectroscopy. Magnetic Reson. Med. 41, 657–663. 10.1002/(sici)1522-2594(199904)41:4<657::aid-mrm3>3.0.co;2-i 10332840

[B12] Neubauer S. (2007). The failing heart — an engine out of fuel. N. Engl. J. Med. 356, 1140–1151. 10.1056/NEJMra063052 17360992

[B13] Purvis L. A. B. Clarke W. T. Biasiolli L. Valkovič L. Robson M. D. Rodgers C. T. (2017). OXSA: an open-source magnetic resonance spectroscopy analysis toolbox in MATLAB. PLOS ONE 12, e0185356. 10.1371/journal.pone.0185356 28938003 PMC5609763

[B14] Robson M. D. Tyler D. J. Neubauer S. (2005). Ultrashort TE chemical shift imaging (UTE-CSI). Magnetic Reson. Med. 53, 267–274. 10.1002/mrm.20344 15678544

[B15] Rodgers C. T. Clarke W. T. Snyder C. Vaughan J. T. Neubauer S. Robson M. D. (2014). Human cardiac 31P magnetic resonance spectroscopy at 7 Tesla. Magnetic Reson. Med. 72, 304–315. 10.1002/mrm.24922 PMC4106879 24006267

[B16] Tyler A. Ellis J. Lau J. Y. C. Miller J. J. Bottomley P. A. Rodgers C. T. (2023). Compartment-based reconstruction of 3D acquisition-weighted 31P cardiac magnetic resonance spectroscopic imaging at 7T: a reproducibility study. NMR Biomed. 36, e4950. 10.1002/nbm.4950 37046414 PMC10658645

[B17] Tyler D. J. Emmanuel Y. Cochlin L. E. Hudsmith L. E. Holloway C. J. Neubauer S. (2009). Reproducibility of 31P cardiac magnetic resonance spectroscopy at 3T. NMR Biomed. 22, 405–413. 10.1002/nbm.1350 19023865

[B18] Tyler D. J. Hudsmith L. E. Clarke K. Neubauer S. Robson M. D. (2008). A comparison of cardiac 31P mrs at 1.5 and 3T. NMR Biomed. 21, 793–798. 10.1002/nbm.1255 18512846

[B19] Valkovič L. Dragonu I. Almujayyaz S. Batzakis A. Young L. A. J. Purvis L. A. B. (2017). Using a whole-body 31P birdcage transmit coil and 16-element receive array for human cardiac metabolic imaging at 7T. PLoS ONE 12, e0187153. 10.1371/journal.pone.0187153 29073228 PMC5658155

[B9] Von Kienlin M. Mejia R. (1991). Spectral localization with optimal pointspread function. J. Magnetic Reson. 94, 268–287. 10.1016/0022-2364(91)90106-4

[B20] Wampl S. Körner T. Valkovič L. Trattnig S. Wolzt M. Meyerspeer M. (2021). Investigating the effect of trigger delay on cardiac 31P mrs signals. Sci. Rep. 11, 9268. 10.1038/s41598-021-87063-8 33927234 PMC8085231

[B21] Zhang Y. Gabr R. E. Zhou J. Weiss R. G. Bottomley P. A. (2013). Highly-accelerated quantitative 2D and 3D localized spectroscopy with linear algebraic modeling (SLAM) and sensitivity encoding. J. Cardiovasc. Magnetic Reson. 237, 125–138. 10.1016/j.jmr.2013.09.018 PMC3976201 24188921

[B22] Zhang Y. Gabr R. E. Schär M. Weiss R. G. Bottomley P. A. (2012). Magnetic resonance spectroscopy with linear algebraic modeling (SLAM) for higher speed and sensitivity. J. Magnetic Reson. 218, 66–76. 10.1016/j.jmr.2012.03.008 PMC3381802 22578557

